# CRY arrests Cop1 to regulate circadian rhythms in mammals

**DOI:** 10.1186/s13008-019-0055-7

**Published:** 2019-11-02

**Authors:** Choogon Lee

**Affiliations:** Department of Biomedical Sciences, Program in Neuroscience, College of Medicine, Florida State University, 1115 West Call Street, Tallahassee, FL 32306 USA

**Keywords:** Circadian rhythms, Transcriptional negative feedback loop, Cryptochrome, CRL4, Cop1, E3 ligase, Glucocorticoids, Glucocorticoid receptor, Gluconeogenesis

## Abstract

Cryptochromes (CRYs) are UVA and blue light photoreceptors present in all major evolutionary lineages ranging from cyanobacteria to plants and animals, including mammals. In plants, blue light activates CRYs to induce photomorphogenesis by inhibiting the CRL4^Cop1^ E3 ligase complex which regulates the degradation of critical transcription factors involved in plant development and growth. However, in mammals, CRYs do not physically interact with Cop1, and of course mammals are not photomorphogenic, leading to the belief that the CRY–Cop1 axis is not conserved in mammals. This belief was recently overturned by Rizzini et al., who showed that although mammalian CRYs do not inhibit Cop1 activity in a light-dependent manner, they antagonize Cop1 activity by displacing Cop1 from CRL4 E3 ligase complex. Because CRYs oscillate, they act in a circadian manner resulting in daily oscillations in Cop1 substrates and the downstream pathways that they regulate. The conserved antagonism of Cop1 by CRY indicates that the CRY–Cop1 axis has an ancient origin, and was repurposed by evolution to regulate photomorphogenesis in plants and circadian rhythms in mammals.

## Background

The Cop1 E3 ubiquitin ligase complex is at the heart of light-mediated signaling pathways that promote development and growth in plants [1, 2]. In both plants and mammals, Cop1 is the substrate-binding subunit in the protein complex that contains other conserved components such as Det1, Ddb1, Cul4 and Rbx1 [3–5]. In darkness, the plant Cop1 is active and targets photomorphogenic transcription factors such as HY5, LAF1 and HFR1 for ubiquitination and degradation. In the presence of blue light, cryptochromes (CRYs) are activated, and CRYs inhibit Cop1 activity resulting in stabilization of those photomorphogenic transcription factors [1, 2]. Although key components are conserved between plant and mammalian CRL4^Cop1^ E3 ligase complexes, there is little overlap in their substrates [1, 6]. Moreover, the mammalian CRYs are core components of the autonomous molecular clock and are not involved in photic entrainment of the clock, unlike CRYs in plants and insects [7, 8].

Circadian rhythms in mammalian physiology and behavior, such as wake–sleep cycles, are governed by a molecular circuit called the circadian clock [7, 9, 10]. The backbone of the circadian clock is a transcriptional negative feedback loop with interacting positive and negative elements [11, 12]. CLOCK (or NPAS2) and BMAL1 are the positive elements, activating transcription of diverse clock-controlled genes (ccgs), including many transcription factors and metabolic genes, as well as the main negative elements, *Period* (*Per*, including *Per1* and *Per2*) and *Cryptochrome* (*Cry*, including *Cry1* and *Cry2*). In the feedback loop, PER and CRY are complexed along with Casein Kinase 1δ and ε (CK1δ/ε) to form an inhibitory complex [11, 12]. Among all essential clock components, PER is the stoichiometrically rate-limiting component; oscillations in PER drive rhythmic inhibition of the transcriptional feedback loop [13, 14]. In the inhibitory complex, CRY provides inhibitory activity by recruiting chromatin remodeling molecules such as HDACs and mSin3B [15, 16], while CK1δ/ε generate circadian time cues by regulating temporal nuclear entry and stability of the inhibitory complex [17, 18].

The canonical view of circadian rhythms is that they are generated by the molecular clock at the transcriptional level through the transcriptional negative feedback loop [7]. However, recent studies have revealed the generation of rhythms at posttranscriptional levels including RNA processing/stability and posttranslational mechanisms [19–22]. For example, only 22% of mRNA cycling genes are driven by the oscillating de novo transcriptional activity in mouse liver [19]. This was revealed through RNA-level analysis of the cistrome and transcriptome, which is relatively straightforward. However, it has been technically challenging to unravel the oscillating proteome and interactome genomewide. Abundance or activity of a protein can be regulated in a circadian manner without oscillations in mRNA levels because the protein can be affected by other oscillating, interacting molecules such as kinases and E3 ligases. The circadian transcriptome is highly enriched for metabolic genes, but some of these genes may not exhibit oscillations at the protein level due to their protein stability [19, 23]. In addition, many genes that do not oscillate at the RNA level may oscillate at the protein or activity level because many proteins in metabolic pathways are regulated by oscillating hormones and metabolites. The main advantage of having a circadian clock is about generating phases of oscillations at the right times of the day to separate non-compatible reactions into different time windows and maximize the efficiency of cellular resources by matching timing between activity of pathways and availability of their target molecules. Therefore, a complete understanding of circadian physiology will be possible only when we will understand how activity phases of clock and their ccgs are established by posttranslational mechanisms.

## Discussion

Stability is a major determining factor in setting amplitude and phase of protein and mRNA oscillations which in turn define the robustness of circadian rhythms. Many genes do not oscillate at mRNA levels, even though their transcription is modulated in a circadian manner at the de novo transcription level, because their mRNA is probably too stable [19]. Similarly, the majority of essential clock genes exhibit robust circadian oscillations at mRNA levels, yet the amplitude of their protein rhythms varies dramatically because their half-lives are all different [13, 24]. PER is the clock protein with the highest turnover rate and, in fact, it exhibits the most dramatic oscillations among all clock proteins, even though the mRNA levels for *Bmal1* and *Cry1* show oscillations as dramatic as that of *Per.*

Several E3 ubiquitin ligases have been implicated as essential clock components because they regulate the half-lives of the core clock proteins, CLOCK, BMAL1, CRY and PER [24–28]. For example, in the absence of SCF^βTrcp1^ and SCF^βTrcp2^, two redundant E3 ligases for PER ubiquitination and degradation, the molecular clock stops functioning because the rate-limiting PER does not oscillate anymore [24]. Although numerous studies have demonstrated how the ubiquitin–proteasome system (UPS) can affect the stability of core clock proteins and thus affect clock function, little is known about how the core clock proteins could affect UPS. To my knowledge, the study led by Rizzini et al. [29] is the first case showing that the core clock components regulate UPS at the posttranslational level, thus showing that circadian regulation of E3 ligases at the posttranslational level could play important roles in circadian physiology. Each E3 ligase usually regulates the stability of multiple substrates, and some of them must be precisely regulated because they are at nodal points of critical pathways.

**Fig. 1:**
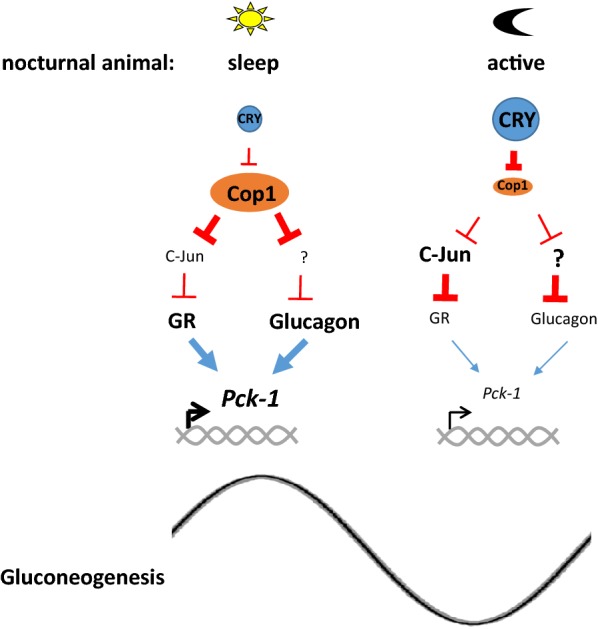
CRY–Cop1 axis regulates circadian physiology at the posttranslational level in mammals. When nocturnal animals sleep during daytime, CRY levels are low in peripheral tissues such as liver, thus minimally inhibiting the Cop1 E3 ligase resulting in destabilization of Cop1 substrates such as c-Jun and other unknown substrates (indicated by “?”). CRY levels slowly increase and peak during night, which will maximally inhibit Cop1 activity during night. This will allow accumulation of Cop1 substrates. For Pck-1, a rate-limiting enzyme for gluconeogenesis, its transcription will be upregulated during daytime when GR and Glucagon signaling are not highly counteracted by inhibitors such as c-Jun and unknown substrate(s) of Cop1. In contrast, *Pck*-*1* transcription is low during the night because these inhibitors accumulate at their peak levels

Because the CRY–Cop1 axis is critical for plant physiology and both components are conserved in mammals, Rizzini et al. tested the hypothesis that CRY functionally regulates Cop1 activity in mammals, even in the absence of the CRY–Cop1 physical interaction that is present in plants. The authors first tested if Cop1 is involved in the CRY-regulated glucocorticoid receptor (GR) transcriptional network. Glucocorticoid production and signaling are regulated by the circadian clock at transcriptional and posttranslational levels [30, 31]. The Evans and Lamia groups rigorously demonstrated that CRYs inhibit transcriptional activity of many nuclear receptors (NRs) including GR by direct protein–protein interaction between CRY and NRs [31, 32]. However, Rizzini et al. hypothesized that CRY inhibition of GR signaling could be mediated by the potentially conserved CRY–Cop1 axis. Consistent with the hypothesis, the authors observed that dexamethasone-induced GR signaling was inhibited significantly when CRY is overexpressed or Cop1 is knocked down in a well-established cell culture model (mouse embryonic fibroblasts, or MEFs). CRY inhibition of GR was dependent on Cop1. When Cop1 is knocked down, CRY overexpression did not significantly inhibit transcription of GR downstream genes in either wt or *Cry1/2* ko cells suggesting that CRY mediates the inhibition through Cop1 similar to the CRY–Cop1 pathway in plants. Interestingly, not all of dexamethasone-induced GR downstream genes were regulated by CRY–Cop1 pathway. Motif enrichment analysis revealed that the affected genes identified by RNA-seq were highly enriched for AP-1 transcription factor binding sites in their promoters. Because c-Jun forms AP-1 complexes with Fos family members, which inhibits GR-mediated transcription, and c-Jun is a well characterized substrate of Cop1 [3, 33–35], the authors proposed that CRY–Cop1 axis is functional in mammalian system and is responsible for transrepression of dexamethasone-induced transcription by GR through the regulation of c-Jun stability. These data can explain why CRYs only affect a subset of GR downstream genes and do not interfere with the GR-regulated NF-κB inflammatory gene network. Glucocorticoids affect diverse aspects of physiology through GR activation and are used to repress over-reactive inflammation because they can repress pro-inflammatory genes including NF-κB downstream genes [36]. Lamia et al. [31] showed that CRYs repress dexamethasone-induced activation of *Pck*-*1* transcription (a rate-limiting enzyme in gluconeogenesis), but did not interfere with dexamethasone-repressed inflammatory genes such as *Tnfα* and *Ccl4*. Because c-Jun does not regulate NF-κB, the data from Rizzini et al. can explain why CRYs only affect a subset of GR downstream genes, which do not include those regulated by NF-κB. The authors further showed that CRY–Cop1 axis may affect pathways other than GR because other canonical substrates of Cop1, such as Ets-1 and p53 are also stabilized by CRY inhibition of Cop1. They speculated that CRY–Cop1 axis may regulate many other important pathways in diverse tissues. Consistent with this speculation, they showed that the CRY–Cop1 pathway also inhibits *Pck*-*1* transcription induced by glucagon signaling, not by GR, in the liver (Fig. 1).

## Conclusions

Circadian clocks allow organisms to anticipate daily changes in the environment by generating diverse phases in activity of important signaling pathways. Transcriptome analysis in different tissues indicated that many genes are expressed in a circadian and tissue-specific manner. Temporal phases of these transcripts are widely distributed throughout a 24-h period, suggesting that many signaling pathways have unique temporal phases in a tissue-specific manner. However, temporal transcript phases of some of the oscillating genes may not correlate well with temporal activity of the signaling pathway they control. For example, a maximum CRY inhibition of GR-induced *Pck*-*1* transcription correlates with peak times of CRY proteins, not those of *Cry* transcripts. Because physiology is ultimately regulated by proteins not by transcripts in most cases, understanding circadian mechanisms at the posttranslational level would give the clearest insights into why temporal regulation of a specific circadian pathway is significant in overall physiology and why it is an advantage to have that unique phase in relation to other pathways.

## Data Availability

Not applicable.

## Abbreviations

Cop1: Constitutive Photomorphogenic 1
CRL4: Cullin4-Ring E3 ligase
HY5: Elongated Hypocotyl 5
LAF1: Long After Far-Red Light 1
HFR1: Long Hypocotyl In Far Red 1
Pck-1: Phosphoenolpyruvate Carboxykinase 1

## References

[1] Lau OS, Deng XW (2012). The photomorphogenic repressors COP1 and DET1: 20 years later. Trends Plant Sci.

[2] Yang HQ, Tang RH, Cashmore AR (2001). The signaling mechanism of *Arabidopsis* CRY1 involves direct interaction with COP1. Plant Cell.

[3] Wertz IE, O’Rourke KM, Zhang Z, Dornan D, Arnott D, Deshaies RJ (2004). Human De-etiolated-1 regulates c-Jun by assembling a CUL4A ubiquitin ligase. Science.

[4] Olma MH, Roy M, Le Bihan T, Sumara I, Maerki S, Larsen B (2009). An interaction network of the mammalian COP9 signalosome identifies Dda1 as a core subunit of multiple Cul4-based E3 ligases. J Cell Sci.

[5] Chen H, Huang X, Gusmaroli G, Terzaghi W, Lau OS, Yanagawa Y (2010). Arabidopsis CULLIN4-damaged DNA binding protein 1 interacts with CONSTITUTIVELY PHOTOMORPHOGENIC1-SUPPRESSOR OF PHYA complexes to regulate photomorphogenesis and flowering time. Plant Cell.

[6] Wei W, Kaelin WG (2011). Good COP1 or bad COP1? In vivo veritas. J Clin Investig.

[7] Reppert SM, Weaver DR (2002). Coordination of circadian timing in mammals. Nature.

[8] Cashmore AR (2003). Cryptochromes: enabling plants and animals to determine circadian time. Cell.

[9] Partch CL, Green CB, Takahashi JS (2014). Molecular architecture of the mammalian circadian clock. Trends Cell Biol.

[10] Bass J, Lazar MA (2016). Circadian time signatures of fitness and disease. Science.

[11] Lowrey PL, Takahashi JS (2011). Genetics of circadian rhythms in Mammalian model organisms. Adv Genet.

[12] Dibner C, Schibler U, Albrecht U (2010). The mammalian circadian timing system: organization and coordination of central and peripheral clocks. Annu Rev Physiol.

[13] Lee C, Etchegaray JP, Cagampang FR, Loudon AS, Reppert SM (2001). Posttranslational mechanisms regulate the mammalian circadian clock. Cell.

[14] Chen R, Schirmer A, Lee Y, Lee H, Kumar V, Yoo SH (2009). Rhythmic PER abundance defines a critical nodal point for negative feedback within the circadian clock mechanism. Mol Cell.

[15] Naruse Y, Oh-hashi K, Iijima N, Naruse M, Yoshioka H, Tanaka M (2004). Circadian and light-induced transcription of clock gene Per1 depends on histone acetylation and deacetylation. Mol Cell Biol.

[16] Shi G, Xie P, Qu Z, Zhang Z, Dong Z, An Y (2016). Distinct roles of HDAC3 in the core circadian negative feedback loop are critical for clock function. Cell Rep.

[17] Lee HM, Chen R, Kim H, Etchegaray JP, Weaver DR, Lee C (2011). The period of the circadian oscillator is primarily determined by the balance between casein kinase 1 and protein phosphatase 1. Proc Natl Acad Sci USA.

[18] Lee H, Chen R, Lee Y, Yoo S, Lee C (2009). Essential roles of CKIdelta and CKIepsilon in the mammalian circadian clock. Proc Natl Acad Sci USA.

[19] Koike N, Yoo SH, Huang HC, Kumar V, Lee C, Kim TK (2012). Transcriptional architecture and chromatin landscape of the core circadian clock in mammals. Science.

[20] So WV, Rosbash M (1997). Post-transcriptional regulation contributes to Drosophila clock gene mRNA cycling. EMBO J.

[21] Baggs JE, Green CB (2003). Nocturnin, a deadenylase in *Xenopus laevis* retina: a mechanism for posttranscriptional control of circadian-related mRNA. Curr Biol.

[22] Chen R, D’Alessandro M, Lee C (2013). miRNAs are required for generating a time delay critical for the circadian oscillator. Curr Biol.

[23] Panda S, Antoch MP, Miller BH, Su AI, Schook AB, Straume M (2002). Coordinated transcription of key pathways in the mouse by the circadian clock. Cell.

[24] D’Alessandro M, Beesley S, Kim JK, Jones Z, Chen R, Wi J (2017). Stability of wake–sleep cycles requires Robust degradation of the PERIOD protein. Curr Biol.

[25] Busino L, Bassermann F, Maiolica A, Lee C, Nolan PM, Godinho SI (2007). SCFFbxl3 controls the oscillation of the circadian clock by directing the degradation of cryptochrome proteins. Science.

[26] Yoo SH, Mohawk JA, Siepka SM, Shan Y, Huh SK, Hong HK (2013). Competing E3 ubiquitin ligases govern circadian periodicity by degradation of CRY in nucleus and cytoplasm. Cell.

[27] Gossan NC, Zhang F, Guo B, Jin D, Yoshitane H, Yao A (2014). The E3 ubiquitin ligase UBE3A is an integral component of the molecular circadian clock through regulating the BMAL1 transcription factor. Nucleic Acids Res.

[28] Lamaze A, Lamouroux A, Vias C, Hung HC, Weber F, Rouyer F (2011). The E3 ubiquitin ligase CTRIP controls CLOCK levels and PERIOD oscillations in Drosophila. EMBO Rep.

[29] Rizzini L, Levine DC, Perelis M, Bass J, Peek CB, Pagano M (2019). Cryptochromes-mediated inhibition of the CRL4(Cop1)-complex assembly defines an evolutionary conserved signaling mechanism. Curr Biol.

[30] Oster H, Damerow S, Kiessling S, Jakubcakova V, Abraham D, Tian J (2006). The circadian rhythm of glucocorticoids is regulated by a gating mechanism residing in the adrenal cortical clock. Cell Metab.

[31] Lamia KA, Papp SJ, Yu RT, Barish GD, Uhlenhaut NH, Jonker JW (2011). Cryptochromes mediate rhythmic repression of the glucocorticoid receptor. Nature.

[32] Kriebs A, Jordan SD, Soto E, Henriksson E, Sandate CR, Vaughan ME (2017). Circadian repressors CRY1 and CRY2 broadly interact with nuclear receptors and modulate transcriptional activity. Proc Natl Acad Sci USA.

[33] Jonat C, Rahmsdorf HJ, Park KK, Cato AC, Gebel S, Ponta H (1990). Antitumor promotion and antiinflammation: down-modulation of AP-1 (Fos/Jun) activity by glucocorticoid hormone. Cell.

[34] Schule R, Rangarajan P, Kliewer S, Ransone LJ, Bolado J, Yang N (1990). Functional antagonism between oncoprotein c-Jun and the glucocorticoid receptor. Cell.

[35] Yang-Yen HF, Chambard JC, Sun YL, Smeal T, Schmidt TJ, Drouin J (1990). Transcriptional interference between c-Jun and the glucocorticoid receptor: mutual inhibition of DNA binding due to direct protein-protein interaction. Cell.

[36] Bamberger CM, Schulte HM, Chrousos GP (1996). Molecular determinants of glucocorticoid receptor function and tissue sensitivity to glucocorticoids. Endocr Rev.

